# Omarigliptin decreases inflammation and insulin resistance in a pleiotropic manner in patients with type 2 diabetes

**DOI:** 10.1186/s13098-020-00533-3

**Published:** 2020-03-24

**Authors:** Sachiko Hattori

**Affiliations:** Department of Endocrinology and Metabolism, Tohto Clinic, 4-1 Kioi-Cho, Chiyoda-Ku, Tokyo, 102-0094 Japan

**Keywords:** Omarigliptin, Once-weekly DPP4 inhibitor, High-sensitivity C-reactive protein, Insulin resistance

## Abstract

**Background:**

Omarigliptin is a potent, selective, oral dipeptidyl peptidase 4 (DPP4) inhibitor with a half-life that allows weekly dosing. Inflammation or insulin resistance might be pathological mediators of cardiovascular events in patients with type 2 diabetes.

**Methods:**

Whether omarigliptin has anti-inflammatory effects that result in decreased levels of high-sensitivity C-reactive protein (hsCRP) and anti-insulin resistance effects that decrease levels of homeostatic model assessment of insulin resistance (HOMA-IR) were investigated. Patients were allocated to continue with daily DPP4 inhibitors (control, n = 28) or to switch from daily DPP4 inhibitors to weekly omarigliptin (omarigliptin, n = 56). Fasting blood and urine samples were collected before, and every 3 months after intervention for 1 year.

**Results:**

Omarigliptin tended to elicit reductions in fasting blood glucose (FBG), LDL-cholesterol, triglyceride, aspartate aminotransferase (AST), alanine aminotransferase (ALT), gamma-glutamyl transpeptidase (γ-GTP), the urinary albumin-to-creatinine ratio (ACR) with logarithmic transformation (log ACR), and systolic and diastolic blood pressure, but the differences did not reach statistical significance compared with control. Values for HDL-cholesterol tended to increase, but also did not reach statistical significance compared with control. Omarigliptin significantly decreased HOMA-IR, remnant-like particle cholesterol (RLP-C), and hsCRP with logarithmic transformation (log hsCRP) compared with control. However, omarigliptin did not affect hemoglobin A1c (HbA1c), body mass index (BMI), and estimated glomerular filtration rates (eGFR).

**Conclusion:**

Omarigliptin decreased inflammation and insulin resistance without affecting HbA1c or BMI. Although how DPP4 inhibitors affect cardiovascular (CV) outcomes remains uncertain, omarigliptin might confer CV benefits at least in part, via pleiotropic anti-inflammatory or anti-insulin resistance effects.

*Trial registration* UMIN Clinical Registry (UMIN000029288). Registered 22 September, 2017, https://upload.umin.ac.jp/UMIN000029288

## Background

Omarigliptin is a potent, selective, oral dipeptidyl peptidase 4 (DPP4) inhibitor with a half-life that allows weekly dosing [1, 2]. Because they can ameliorate glycemic control in patients with type 2 diabetes without severe side effects, daily DPP-4 inhibitors have become an established part of the treatment regimens for patients over the past 10 years. Oral dipeptidyl peptidase 4 (DPP4) inhibitors increase pancreatic β-cells and insulin sensitivity in the liver, muscle and adipose tissues [3]. Besides, DPP4 therapy has anti-inflammatory and anti-atherogenic effects, and can improve endothelial function and reduce vascular stiffness [3]. However, daily doses of DPP4 inhibitors do not lower plasma insulin in patients with insulin resistance [4–6]. In addition, whether weekly doses of omarigliptin can reduce inflammation or insulin resistance remains unknown. We aimed to determine whether inflammation and insulin resistance can be decreased more effectively by omarigliptin 25 mg/week than by sitagliptin 50 mg/day or linagliptin 5 mg/day for 12 months.

## Methods

### Study design and participants

This single-center, open-label, randomized, prospective study included 84 patients who have attended our clinic for at least 12 months and had hemoglobin A1c (HbA1c) > 6.0% regardless of diet, exercise, and daily medication with the DPP4 inhibitors sitagliptin (50 mg) or linagliptin (5 mg). Table 1 shows the inclusion and exclusion criteria. The patients were allocated in a 1:2 ratio using numbered containers to continue the same daily regimens of sitagliptin 50 mg (n = 19) or linagliptin 5 mg (n = 9) as a control group (n = 28) or to switch from these inhibitors (n = 40 and n = 16, respectively) to omarigliptin 25 mg/week (omarigliptin group: n = 56). Table 2 shows that all patients continued medication with oral hypoglycemic drugs (sulfonylureas, metformin, or an α-glucosidase inhibitor), antihypertensive (angiotensin II receptor blockers or calcium channel blockers), and antihyperlipidemic agents (statins or fibrates). The patients were given detailed explanations of the study protocol, then all patients provided written informed consent to participate in the study. The study protocol was approved by the Ethics Committee at Tohto Clinic. This trial was registered with the University Hospital Medical Information Network (UMIN000029288).

**Table 1:** Entry criteria and exclusion criteria

Entry criteria
(i) Age ≥ 20 years
(ii) Type 2 diabetes mellitus with HbA1c > 6.0%
(iii) Body mass index (BMI) more than 20 and less than 30
(iv) Treatment with diet, exercise therapy and daily DPP4 inhibitors
Exclusion criteria
(i) Type 1 diabetes
(ii) Severe diabetic metabolic complications, such as ketoacidosis
(iii) Severe liver dysfunction
(iv) Pregnant or breast‐feeding women and those who might be pregnant

**Table 2:** Baseline characteristics and medications of the participants

	Control (n = 28)	Omarigliptin (n = 56)	p value
Age (years)	59.17 ± 7.85	59.00 ± 7.33	0.638
Male/(female)	21 (7)	40 (16)	0.8
Baseline medication			
Sitagliptin	19	40	0.802
Linagliptin	9	16	0.802
Sulfonylureas	5	11	1
Metformin	7	14	1
α-GI	4	6	0.7248
ARB	9	18	1
CCB	6	14	0.7913
Statins	9	19	1
Fibrates	5	11	1

Data were expressed as mean ± standard deviation

α-GI, α-glicosidase inhibitor; ARB, angiotensin II receptor blocker; CCB, calcium channel blocker

### Measurements and endpoints

Blood and urine samples were collected from all included patients after an overnight fast at baseline and then at intervals of 3 months for 1 year. Values for high-sensitivity C-reactive protein (hsCRP), immunoreactive insulin (IRI), remnant-like particle cholesterol (RLP-C), and urinary albumin were assessed at LSI Medicine Corporation (Tokyo, Japan). Other biochemical data were generated in-house. The homeostatic model assessment of insulin resistance (HOMA-IR) was calculated as (fasting blood glucose (FBG) × IRI)/450. The primary and secondary endpoints were changes among HbA1c, BMI, hsCRP, and HOMA-IR between baseline and 1 year later, and between baseline and 3-month intervals in the control and omarigliptin groups.

### Statistical analysis

Data are expressed as mean ± standard deviation (SD) and all data were statistically analyzed using EZR software version 1.21 [7]. Parameters before, and at 3, 6, 9, and 12 months after treatment were compared using paired t tests. Differences in parameters between the control and omarigliptin groups over 12 months were analyzed using repeated measures ANOVA. Categorical and continuous baseline values and medications were analyzed using Fisher exact tests and t tests, respectively. Statistical significance for differences was set at p < 0.05.

## Results

**Fig. 1:**
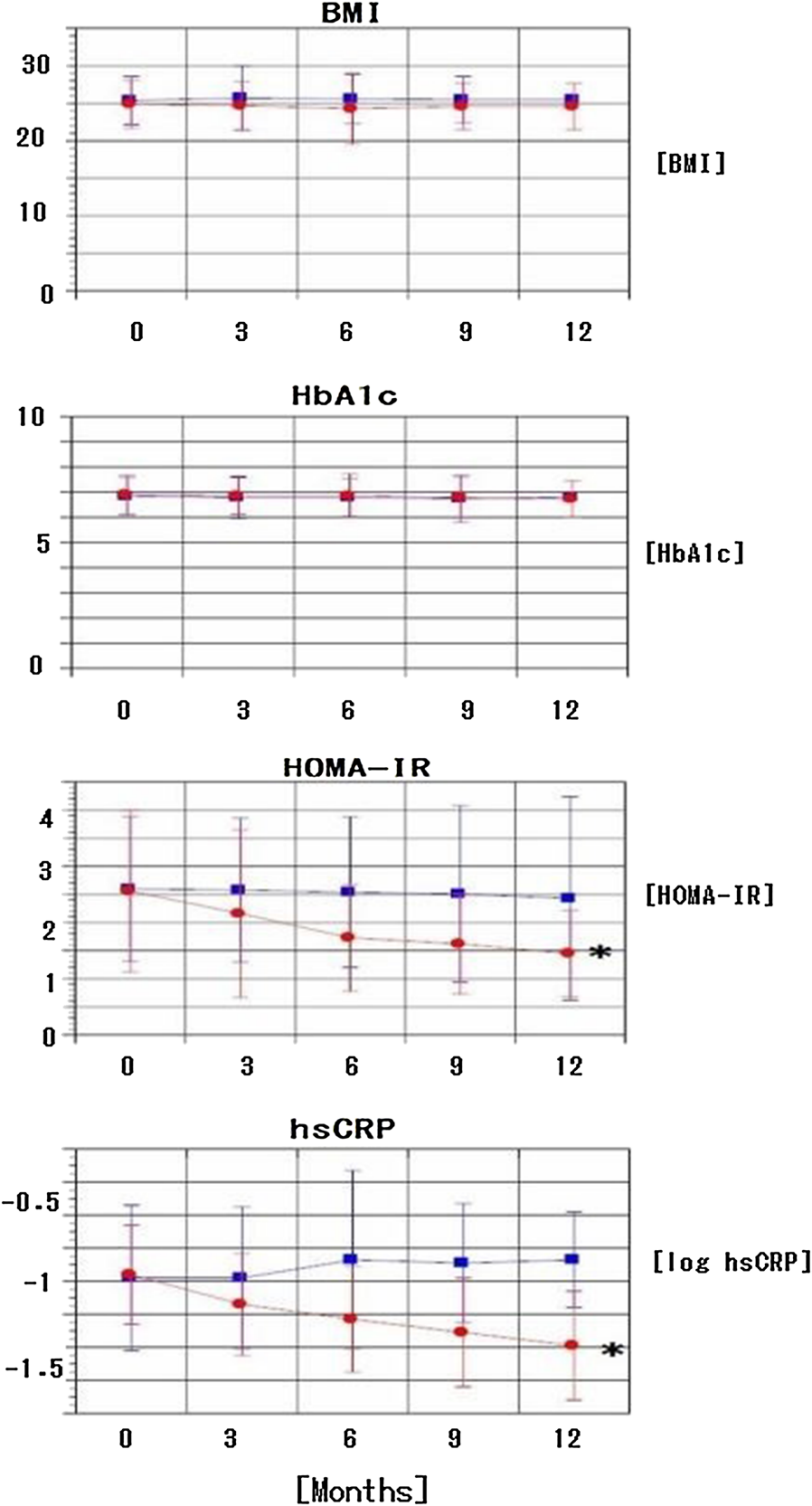
Time course of HbA1c, BMI, HOMA-IR, and log hsCRP in patients treated with daily DPP4 inhibitors and weekly omarigliptin. Red circles, omarigliptin; blue squares, daily DPP4 inhibitors (control group). Data are presented as mean ± SD. Group differences between control and omarigliptin were analyzed using repeated measures ANOVA (*p < 0.05, p = 0.73536, 0.2176, 0.01409, and 0.00712 for HbA1c, BMI, HOMA-IR, and log hsCRP, respectively)

All examined parameters remained unaltered in the control group for 12 months (Table 3). Omarigliptin slightly, but not significantly reduced FBG values, but significantly reduced IRI and HOMA-IR values compared with the control. Values for HbA1c, BMI, and estimated glomerular filtration rates (eGFR) remained essentially unchanged in the omarigliptin group for 12 months. Omarigliptin substantially reduced RLP-C and hsCRP with logarithmic transformation, and reduced values for aspartate aminotransferase (AST), alanine aminotransferase (ALT), gamma-glutamyl transpeptidase (γ-GTP), the urine albumin-to-creatinine ratio with logarithmic transformation (log ACR), LDL-cholesterol, HDL-cholesterol, triglycerides (TG), and systolic and diastolic blood pressure slightly, but not significantly, compared with the control (Tables 3 and 4). Figure 1 shows the time courses for HbA1c, BMI, hsCRP with logarithmic transformation, and HOMA-IR. Omarigliptin substantially decreased inflammation and insulin resistance without affecting the control of glucose levels and body weight.

**Table 3:** Clinical parameters of patients treated with daily DPP4 inhibitors or weekly omarigliptin

Month of study	Control (n = 28)					Omarigliptin (n = 56)				
	0	3	6	9	12	0	3	6	9	12
FBG (mg/dl)	130.8 (24.8)	129.5 (22.9)	129.8 (28.8)	127.8 (30.6)	129.0 (26.8)	133.8 (19.1)	129.6 (24.6)*	123.7 (29.3)*	123.6 (18.1)*	117.1 (18.7)*
IRI (μU/ml)	7.12 (1.87)	7.15 (3.02)	7.26 (3.05)	7.21(2.49)	7.31 (3.32)	7.37 (3.7)	6.40 (3.14)*	5.56 (2.71)*	5.21(2.3)*	4.72 (2.12)*
HOMA-IR	2.60 (1.29)	2.58 (1.28)	2.54 (1.34)	2.51 (1.57)	2.43 (1.81)	2.56 (1.44)	2.16 (1.49)*	1.73 (0.95)*	1.62 (0.89)*	1.45 (0.77)*
HbA1c (%)	6.85 (0.75)	6.79 (0.84)	6.79 (0.75)	6.75 (0.93)	6.81(0.79)	6.91 (0.77)	6.86 (0.73)	6.86 (0.87)	6.79 (0.81)*	6.73 (0.72)*
BMI	25.4 (3.2)	25.7 (4.3)	25.6 (3.3)	25.5 (3.1)	25.5 (3.7)	24.9 (3.2)	24.7 (3.2)	24.3 (4.7)	24.6 (3.1)	24.6 (3.1)
log (hsCRP)	− 0.98 (0.44)	− 0.98 (0.43)	− 0.87 (0.54)	− 0.89 (0.36)	− 0.87 (0.29)	− 0.96 (0.30)	− 1.14 (0.31)*	− 1.23 (0.32)*	− 1.31 (0.33)*	− 1.39 (0.33)*
LDL-C (mg/dl)	103.6 (24.1)	101.2 (23.9)	101.1 (25.8)	101.9 (24.5)	103.8 (27.2)	102.8 (23.6)	102.0 (24.7)	102.5 (23.6)	98.6 (26.8)	95.7 (25.9)*
HDL-C (mg/dl)	57.1 (12.9)	56.5 (11.8)	56.7 (13.7)	57.5 (12.1)	55.4 (12.3)	57.3 (12.8)	57.8 (14.9)	57.3 (12.8)	57.8 (12.6)	56.2 (16.2)
TG (mg/dl)	129.4 (61.3)	139.8 (81.7)	142.6 (64.1)	139.9 (58.4)	153.5 (75.0)	124.1(68.5)	121.6 (53.3)	114.3 (50.0)	106.0 (45.5)*	103.1 (33.7)*
RLP-C (mg/dl)	4.77 (3.66)	4.74 (3.65)	4.72 (3.28)	4.61 (3.13)	4.51 (2.57)	4.78 (3.19)	4.34 (2.15)*	3.73 (1.78)*	3.36 (1.49)*	2.91 (0.94)*
AST (IU/L)	25.6 (14.6)	25.2 (8.1)	25.4 (7.7)	25.1 (5.9)	25.7 (6.9)	24.4 (10.5)	22.6 (7.1)*	22.7 (7.8)	22.8 (8.4)	22.3 (6.6)
ALT (IU/L)	25.2 (12.6)	25.3 (14.6)	24.4 (16.2)	24.3 (17.3)	25.7 (14.6)	25.0 (15.5)	23.1 (11.7)	23.6 (12.2)	23.1 (10.8)	21.6 (10.9)*
γGTP (IU/L)	40.1 (35.6)	39.5 (32.4)	40.7 (33.6)	38.5 (29.7)	38.3 (30.8)	38.8 (33.7)	38.6 (34.0)	35.7 (25.4)	33.8 (33.7)	31.5 (19.8)*
eGFR (mL/min/1.73m2)	69.4 (16.7)	68.5 (18.8)	69.6 (18.2)	69.7 (15.6)	69.7 (17.5)	68.0 (13.1)	68.2 (13.3)	68.2 (14.5)	67.9 (14.1)	67.8 (13.4)*
log (ACR)	1.18 (0.59)	1.20 (0.51)	1.23 (0.56)	1.27 (0.59)	1.27 (0.44)	1.27 (0.56)	1.13 (0.58)*	1.18 (0.56)	1.17 (0.56)	1.12 (0.60)*
SBP (mmHg)	125.0 (15.8)	127.0 (18.4)	126.6 (17.5)	125.1 (18.5)	124.7 (16.6)	123.7 (14.3)	119.5 (13.0)*	121.4 (12.3)	119.8 (12.3)*	118.4 (12.9)*
DBP (mmHg)	70.5 (10.3)	70.8 (9.3)	70.1 (9.7)	70.2 (10.7)	70.5 (14.2)	70.5 (9.0)	68.4 (9.8)	69.6 (8.7)	68.2 (8.3)	67.9 (9.0)*

Values are shown as mean ± SD in parentheses

FBG, fasting blood glucose; IRI, immunoreactive insulin; HOMA-IR, homeostatic model assessment of insulin resistance; HbA1c, hemoglobin, A1c; BMI, body mass index; hsCRP, high sensitivity C-reactive protein; LDL-C, low-density lipoprotein cholesterol; HDL-C, high-density, lipoprotein cholesterol; TG, triglyceride; RLP-C, remnant-like particle cholesterol; AST, aspartate aminotransferase; ALT, alanine, aminotransferase; γGTP, gamma-glutamyl transpeptidase; eGFR, estimated glomerular filtration rate; ACR, albumin-to-creatinine ratio; SBP, systolic blood pressure; DBP, diastolic blood pressure

* Intragroup comparison: p < 0.05 (paired t test)

**Table 4:** Comparison of clinical parameters between patients treated with daily DPP4 inhibitors or weekly omarigliptin

	FBG	IRI	HOMA-IR	HbA1c	BMI	log(hsCRP)	LDL-C	HDL-C	TG	RLP-C	AST	ALT	γGTP	eGFR	log(ACR)	SBP	DBP
Factor1.C.M	0.0822	0.0445*	0.0140*	0.735	0.217	0.00712*	0.0518	0.0579	0.0571	0.00701*	0.0593	0.0501	0.0627	0.673	0.588	0.0553	0.0818
Time	0.0318*	0.2124	0.0526	0.119	0.85	0.00077*	0.969	0.272	0.909	0.315	0.243	0.694	0.671	0.675	0.0235*	0.493	0.137
Factor1.C.M:Time	< 0.001*	< 0.001*	< 0.001*	0.029	0.465	< 0.001*	0.0891	0.476	0.0174*	< 0.001*	0.978	0.68	0.010*	0.531	< 0.001*	0.0707	0.741

FBG, fasting blood glucose; IRI, immunoreactive insulin; HOMA-IR, homeostatic model assessment of insulin resistance; HbA1c, hemoglobin, A1c; BMI, body mass index; hsCRP, high sensitivity C-reactive protein; LDL-C, low-density lipoprotein choresterol; HDL-C, high-density, lipoprotein cholesterol; TG, triglyceride; RLP-C, remnant-like particle cholesterol; AST, aspartate aminotransferase; ALT, alanine, aminotransferase; γGTP, gamma-glutamyl transpeptidase; eGFR, estimated glomerular filtration rate; ACR, albumin-to-creatinine ratio; SBP, systolic blood pressure; DBP, diastolic blood pressure

p values were presented

Factor 1 C.M (between-subject factor): group difference

Time (within-subject factor): time-dependent difference

Factor 1 C.M.: Time: interaction

Comparison for 12 months between patients treated with daily DPP4 inhibitors and weekly omarigliptin

* p < 0.05 with repeated measures ANOVA

## Discussion

Omarigliptin is a potent, selective, DPP4 inhibitor that is administered weekly, is generally well tolerated and associated with a very low incidence of adverse events [8, 9]. No adverse events including mild hypoglycemia developed in either the omarigliptin group or the control group in the present study.

This study aimed to determine whether weekly omarigliptin could improve inflammation and insulin resistance more effectively than daily sitagliptin and linagliptin. The results showed that weekly doses of omarigliptin decreased hsCRP and remnant lipoproteins and ameliorated insulin resistance more effectively than daily doses of the other two DPP4 inhibitors.

Daily doses of DPP4 inhibitors do not result in decreased plasma insulin levels or ameliorated insulin resistance in insulin-resistant, hyperinsulinemic individuals [4–6]. Adipose tissue inflammation and insulin resistance closely correlate in obese persons and blocking systemic or adipose tissue macrophage inflammation improves insulin sensitivity in obese mice [10–13]. A recent study has shown that DPP4 secreted by hepatocytes in obese mice promotes adipose inflammation and insulin resistance [14]. Omarigliptin is a DPP4 inhibitor with a half-life that enables weekly dosing and it has a wide organ distribution including the liver [1, 15]. Therefore, it might decrease morning glucagon levels more effectively than daily DPP4 inhibitors [16]. However, we did not measure values for glucagon, although it can lower FBG and insulin levels, and consequently decrease HOMA-IR. Omarigliptin might also decrease DPP4 secretion by hepatocytes that might promote adipose inflammation and insulin resistance. We found that omarigliptin tended to reduce liver transaminases, which could be due to reducing the amount of fat in the liver that might overexpress DPP4 [17, 18].

Remnant lipoproteins are thought to be atherogenic, and amounts of various remnant lipoproteins circulating in the blood are reflected in remnant-like particle cholesterol (RLP-C). Thus, high RLP-C levels are believed to constitute a significant independent risk factor for coronary artery disease (CAD) and to predict future coronary events in patients with confirmed CAD, as well as those with type 2 diabetes [19]. The present study found that omarigliptin decreases RLP-C levels, which is closely associated with the amelioration of insulin resistance and inflammation [20].

Daily DPP4 inhibitors including omarigliptin do not increase the risk of major adverse cardiovascular events [2]. The present study found that omarigliptin exerted more anti-inflammatory and anti-insulin resistance effects than daily DPP4 inhibitors. Arima et al. associated hsCRP levels with future coronary heart disease events in a general Japanese population [21]. Therefore, a decrease in hsCRP levels caused by omarigliptin might result in considerable CV risk reduction in Japanese patients with type 2 diabetes mellitus. A meta-analysis of the three major cardiovascular outcomes of DPP4 inhibitors by Crowley et al. [22] showed that metformin might modulate the effects of DPP4 inhibitors on CV outcomes. They speculated that the mechanism through which metformin potentiates the effectiveness of DPP4 inhibitors is via endogenous glucagon-like peptide-1 (GLP-1): metformin increases levels of GLP-1, whereas DPP4 inhibitors prevent GLP-1 degradation. Addy et al. showed that although DPP-4 activity was inhibited by ~ 90%, postprandial (PP) 4-h weighted mean active GLP-1 concentrations were increased ~ twofold, and PP glucose was significantly reduced by omarigliptin compared with a placebo in a pooled population [23]. However, GLP-1 levels have not been compared between omarigliptin and other DPP4 inhibitors.

The regulation of glycemia by DPP4 inhibition is mainly achieved by preventing endogenous GLP-1 degradation. However, some glycemia-independent pleiotropic actions of DPP4 inhibitors including antioxidant and neurorestorative effects have been found [24]. Ayoub et al. advocated repositioning omarigliptin as a weekly anti-parkinsonian agent because omarigliptin was the first gliptin to cross the blood brain barrier [25]. These findings suggest that omarigliptin confers pleiotropic pharmacological benefits due to a low molecular weight and lipophilic properties that permit wide organ distribution.

The limitations of the present study were the relatively small patient cohort and the open label design. We repeatedly evaluated the HOMA-IR to determine changes in insulin resistance. Although insulin sensitivity should ideally be precisely evaluated using the glucose clamp procedure [26, 27], this method is impractical for the long-term assessment of large numbers of patients. Nevertheless, the glucose clamp procedure is needed to accurately evaluate the ability of omarigliptin to decrease insulin resistance.

## Conclusion

Daily-DPP4 inhibitors need to inhibit > 80% of DPP4 activity to maximally lower glucose values. The intake of 25 mg of omarigliptin rapidly induces the maximum inhibitory action of DPP4 by ~ 98%, and this is maintained at 83% after 7 days which allows weekly dosing [28]. Here, we showed that weekly doses of omarigliptin ameliorated insulin resistance and decreased hsCRP levels without affecting the control of glucose levels or body weight, compared with daily doses of sitagliptin and linagliptin. Despite uncertainty as to how DPP4 inhibitors affect CV outcomes, omarigliptin might confer CV benefits at least in part, via pleiotropic anti-inflammatory or anti-insulin resistance effects.

## Data Availability

The datasets analyzed during the current study are not publicly available due to some relevant ongoing studies, but may be available from the corresponding author upon reasonable request.

## Abbreviations

ACR: Albumin-to-creatinine ratio
ALT: Alanine aminotransferase
AST: Aspartate aminotransferase
BMI: Body mass index
CAD: Coronary artery disease
CV: Cardiovascular
DBP: Diastolic blood pressure
DPP4: Dipeptidyl peptidase 4
eGFR: Estimated glomerular filtration rate
GLP-1: Glucagon-like peptide-1
FBG: Fasting blood glucose
HbA1c: Hemoglobin A1c
HDL: High-density lipoprotein
HOMA-IR: Homeostatic model assessment of insulin resistance
hsCRP: High sensitivity C-reactive protein
IRI: Immunoreactive insulin
LDL: Low-density lipoprotein
RLP-C: Remnant-like particle cholesterol
SBP: Systolic blood pressure
TG: Triglyceride
γGTP: Gamma-glutamyl transpeptidase
